# The pursuit of intimacy: intimate relationship development for women with physical disabilities

**DOI:** 10.3389/fresc.2023.1070501

**Published:** 2023-08-04

**Authors:** Derek Ruiz, Alexandra Kriofske Mainella, David A. Rosenthal

**Affiliations:** ^1^ Department of Rehabilitation, Disability Counseling, Southern University and A&M College, Baton Rouge, LA, United States; ^2^ Department of Education and Counseling Psychology, Marquette University, Milwaukee, WI, United States; ^3^ Department of Rehabilitation Psychology and Special Education, University of Wisconsin Madison, Madison, WI, United States

**Keywords:** intimacy, disability, sexuality, relationship development, consensual qualitative research, physical disability

## Abstract

**Background and purpose:**

Understanding the barriers and facilitators to intimate relationship development among women with physical disabilities is crucial for promoting positive rehabilitation outcomes. This study investigates these factors using a Consensual Qualitative Research approach.

**Methods:**

The research team utilized Consensual Qualitative Research. Consensual Qualitative Research is widely used in various social services to address limitations inherent limitations of other qualitative methods. Women with various physical disabilities (n=6) were identified and interviewed using a utilized a semi-structured interview. Their attitudes towards intimacy, outcome expectancy, and self-concept of women with physical disabilities were examined. The impact of physical disabilities, including disability stigma, on these factors was also assessed. Data was coded utilizing multiple researcher consensus as well as an auditor to consensually agree upon domains and core ideas while taking into account validity and trustworthiness, as per the methodology chosen.

**Results:**

The study discovered general themes (100% representation) concerning core values and barriers to intimacy, societal barriers to intimate relationships, and hope for future relationships. Typical themes (50-83% representation) included non-romantic intimacy, physical barriers to intimacy, perceived limitations, and negative outcome expectancies. Across the board, the influence of disability limitations, identity, and societal attitudes became evident. The role of rehabilitation professionals in advocating for social skills development and self-confidence enhancement emerged as crucial.

**Conclusions:**

The study illuminated barriers to intimacy among women with physical disabilities, such as societal stigma and self-concept associated with disability. The critical role of rehabilitation professionals in normalizing intimacy discussions and equipping individuals with necessary social skills and self-confidence was underscored. This focus could yield enhanced intimate relationship outcomes and improve the quality of life for women with physical disabilities.

## 1. Introduction

Personal relationships and intimacy enrich our lives as sources of expressing connection, love, joy, creativity, desire, identity, and individuality. Relationships and intimacy develop across the lifespan and provide many opportunities for growth and personal discovery. However, research indicates that some people with disabilities encounter obstacles in developing relationships and exploring and expressing their intimacy and sexuality. Adults with disabilities who seek appropriate intimate relationships are not operating on a level playing field (1).

Developing and maintaining intimate relationships with others may be one of the most meaningful and challenging tasks we undertake in life and substantially impacts our overall happiness and quality of life (2, 3). Physical and mental impairments may alter functioning but do not eliminate the need for intimacy (4, 5). People with disabilities may be significantly disadvantaged in the pursuit of satisfying intimate relationships due to a confluence of reasons, including but not limited to negative societal attitudes and values towards the sexuality of people with disabilities, as well as perceived and actual limitations due to disability (6–8).

Most of the research available on intimate relationship development focuses on people without disabilities, though people with disabilities face unique challenges in developing such relationships (9–11). These difficulties must be better understood to potentially address them with both those who are currently seeking intimate relationships and those who will, in the future, pursue intimacy. This study sought to gain a better understanding of the subjective experiences of women with physical disabilities in developing intimate relationships, understand what aspects of intimacy and sexuality are most important for women with physical disabilities, and identify facilitators and challenges to intimate relationships.

## 2. Literature review

Before the 1970 s, there was little to no research conducted on the sexuality of people with disabilities (6). Since then, the sexuality of people with disabilities has received some attention, but not proportionally to those without disabilities. According to Nosek et al. (1996), (4) the lack of research efforts regarding the sexuality of persons with disabilities reflects a general failure of social and behavioral sciences to identify sexuality as a prominent issue for this population.

### 2.1. Attitudes and stigma about sexuality

Prevalent attitudes about sexuality and disability can lead to the internalization of negative attitudes and beliefs (11, 12). These attitudes and beliefs can sometimes become self-fulfilling prophecies, leading people with disabilities to refrain from intimate relationships become isolated (6, 8, 13). A review of research shows that sexual acts involving people with disabilities are viewed more negatively than in the context of non-disability (6). Research indicates that persons with disabilities are more readily accepted as colleagues and casual friends than dating partners (14). Disability impairs people from having intimate needs met and impairs their capability to express their sexuality. These attitudes can be especially harmful when the global devaluation of people with disabilities results in feelings of diminished worth and desirability. Negative attitudes towards the sexuality of people with disabilities are expressed in many life areas ranging from patronizing behaviors, avoidance, and rejection, to abuse that can be physical, emotional, or sexual (15, 16).

### 2.2. Self-efficacy and outcome expectations

Albert Bandura believed that young people learn about themselves and behavior through observing others (17). The foundational work of Bandura emphasized the importance of self-concept and self-efficacy. Self-concept is the collection of self-schemas and their interactions with self-esteem, self-knowledge, and the social self to form a concept of the self as a whole. Self-concept encompasses past, present, and possible future selves, which may impact certain behaviors (18). Self-concept encompasses many domains including, personal, academic, and sexual. Self-concept usually serves to answer the question of “Who am I?” (19).

Self-concept in general is the sense of positive view of self (20). Within the realm of sexuality, self-concept is an important subject because it may be the starting place for researchers to bridge the gap between sexuality and life satisfaction. People with disabilities are conditioned to think of themselves without sexuality. The sexuality of people with disabilities has not typically been seen as having a significant impact on their quality of life, which may be due to societal removal of that importance (6). People with disabilities, through the experience of stigma, have difficulty with health and wellness concerns, and are conditioned to devalue their own intimacy (21). This has led to gaps in the literature involving the intimacy of people with disabilities and must be corrected.

Also noted among Bandura’s social learning theory is the construct of self-efficacy (20). Self-efficacy is a person’s belief in his or her abilities to complete a given behavior correctly. Outcome expectancy is the expected response after a given behavior. Additionally, environmental aspects influence an individual’s ability to complete the behavior successfully. Imperative to psychosexual development is the observation of modeling from others to develop social schema. Self-efficacy and outcome expectations are built through this modeling and environmental facilitation or hindrance (22). Sexual self-efficacy, much like general self-efficacy, is the belief in abilities to care for sexual health and is correlated with lower risk in sexual decision making and higher sexual and relationship satisfaction (23). One way in which individuals can bolster their self-efficacy in sexual and romantic relationships is to communicate with partners.

Sexual communication is highlighted as one facilitator influencing sexual health behavior (24) Due heavily to myths surrounding the sexuality of people with disabilities, such as asexuality, lack of sex drive, inability to partake in sexual activity, and lack of social judgment (25), people with disabilities have been denied the same psychosexual developable milestones as their non-disabled peers. Studies have shown that people with disabilities are in intimate relationships, but they lack support to address relationship issues including disability related barriers (12).

### 2.3. Importance of intimacy

Intimacy not only plays a critical role in the passage through developmental stages (26) but may also contribute to resistance to diseases and disabling conditions, as well as a lower rate of mental illness (27). Intimacy has been defined in multidimensional, general, and operational terms (28), but people with disabilities are often left out of the research concerning intimate relationships.

## 3. Methodology

This research utilized Consensual Qualitative Research (CQR) as its primary paradigm. Consensual Qualitative Research (CQR) is an inductive form of research that allows results to emerge from the stories and contexts given by participants and is widely used in various social sciences, counseling, and rehabilitation counseling research (29). CQR is primarily based in grounded theory and, by design, specifically addresses some of the limitations inherent in qualitative research (30). CQR utilizes a team of researchers to collaborate and consensually analyze the data and auditors to examine the research team’s work and provide feedback at each step of the process to address threats to validity (31). This methodology also addresses the need for a structured coding method and data analysis lacking in most qualitative research methods. CQR methods are both naturalistic and interactive; meaning is surmised from words and texts, the context of participants’ responses, and interviewers’ interaction with participants through semi-structured interviews, probes, and clarifications (32). Finally, the representativeness of the main ideas is assessed within a CQR methodology, which is not typical of grounded theory, which does not usually state the proportion of shared ideas.

### 3.1. Research team

Qualitative studies utilize researchers as the primary instruments for data collection and analysis. Researcher personal biases, values, and experiences can influence the rigor of research (30). Researchers participating in the study recorded their biases and expectations before interviews, then compared those with the results at various times during data analysis (33). The primary researcher has a rich history of activism concerning human sexuality related to people with disabilities. The other researchers were master’s level Rehabilitation Counseling students who reported intimacy as necessary for people with disabilities to varying degrees. The study auditor was a doctoral student in a Rehabilitation Counselor Education Program with no prior connection to the study and a sexual health educator with experience working closely with individuals with disabilities.

### 3.2. Interview protocol

#### 3.2.1. Development of the protocol

With the help of content experts, the interview protocol was developed from a literature review and personal experience (34). The CQR process calls for open-ended questions that provide a framework for responses, combined with follow-up questions that encourage elaboration of responses while intentionally avoiding influencing responses. The interview was structured with respect to two primary goals rapport building and information gathering. The interview protocol focused on the main topic of interest and focused on the participant’s subjective definition of terms. Scripted questions were used to ensure consistent information and probed specifically for attitudes, beliefs, and feelings about certain experiences. The final section of the interview protocol asked participants to reflect more broadly on issues related to intimate relationship development.

#### 3.2.2. Pilot

The interview protocol was assessed as per Hill and colleagues (32) with one participant who fulfilled participant criteria but not included in the final sample. The pilot run of the protocol provided valuable information through the interview responses and participant feedback and used to revise the final protocol used in the study.

#### 3.2.3. Sample

Women are generally underrepresented in research, specifically rehabilitation research (35). Participants were drawn from two disability service organizations, and from there, a snowball convenience sampling method of initial participants was used to recruit other participants. Six women ranged in age from 23 to 60 years old, currently living independently in the community. Five out of six identified as Caucasian, the other as Black. All participants self-identified as having visible physical disabilities. Three reported having muscular dystrophy, two reported cerebral palsy, and the other reported amniotic band syndrome. The relationship statuses, sexual orientations, and education levels of the participants were mixed and represented a broad range of experiences (Table 1).

**Table 1:** Participant demographic information.

Age	Race	Disability reported	Relationship status	Sexual orientation	Level of education
23	Caucasian	Muscular dystrophy	Single	Bisexual	College
27	Caucasian	Amniotic band syndrome	Single	Heterosexual	College
28	Black	Cerebral palsy	Partnered	Homosexual	Some college
31	Caucasian	Cerebral palsy	Single	Heterosexual	Graduate degree
58	Caucasian	Muscular dystrophy	Partnered	Homosexual	Highschool
60	Caucasian	Muscular dystrophy	Single	Bisexual	College

### 3.3. Data analysis procedures

#### 3.3.1. Coding domains

The transcribed interviews were used to create a list of meaningful and unique topic areas, called domains. Researchers independently reviewed the data and identified proposed domains through an iterative process of identifying and extracting meaningful data units based on excerpts of transcript and general ideas. The team met to compare notes and consensually create an initial domain list that best fit the data (32, 34). The domain list was then given to the auditor to review. The auditor provided feedback about the clarity of the domain titles the level of specificity of the domains. Data was then blocked and assigned to domains. The group came together and formed a consensus on the appropriateness of the block and its domain.

#### 3.3.2. Core ideas

The next step was the construction of core ideas from the existing data units. This allowed for each participant’s response to be in clear, understandable language with consistency across cases. Domain development was done similarly to care ideas; researchers developed core ideas for several cases, then developed core ideas for the remaining cases with consensus from the research team. Consensus yielded a version of the data that contained the raw data, core ideas, and domains. The auditors again reviewed the consensus version and recommended changes, after which the team met to discuss them.

### 3.4. Validity and trustworthiness

Within qualitative research, researchers use the term *trustworthiness* instead of *validity* (36). *Trustworthiness* refers to the researchers’ assertion to have utilized appropriate, adequate, and reliable methods to report the findings correctly. For this research, trustworthiness was assessed by the criteria of Williams and Morrow (37) to include: (1) establishing the integrity of the data, (2) balance of the tension between subjectivity and reflexivity, and (3) communicating findings and their applicability to research and practice. Additionally, researchers used theoretical saturation to establish trustworthiness. *Theoretical saturation* is a point where no new data emerges during a study and the point where the researcher becomes empirically sure of the findings (38). Hill, Thompson, and Williams (1997) (34) refer to this in consensual qualitative research as *stability of findings*.

Finally**,** testimonial validity was achieved through participant review of study findings to ensure researcher interpretations accurately reflected their lived experiences. Participants who chose to participate stated that the findings accurately represented their lived experiences.

## 4. Results

*“We want people to inspire our lives, and we want to inspire other people in their lives too.”*

The women surveyed spoke of many factors that had an impact on their views about intimate relationship development. This analysis produced three distinct domains: Attitudes about Intimacy, Barriers to Intimate Relationship Development, and Outcome Expectancies. Each domain consisted of several listed core-ideas. The representativeness identifiers are labeled as follows: “general” denotes 5 or 6 total participants, “typical” denotes 3 or 4 participants, “variant” denotes 2 participants, and “rare” denotes a single participant. The number of participants whose responses were coded into that particular category is cited in parentheses (Table 2).

**Table 2:** Domain and category data collected. As per consensual qualitative coding convention, identifiers are labeled as follows: “general” denotes 5 or 6 total participants, “typical” denotes 3 or 4 participants, “variant” denotes 2 participants, and “rare” denotes a single participant.

Domain	Category	Representation	*n*	Percent
Attitudes about intimacy				
	Core values	General	6	100%
	Non-Romantic intimacy	Typical	4	67%
	Barriers to intimacy	General	6	100%
Barriers to intimate relationships				
	Societal barriers	General	6	100%
	Physical barriers to intimacy	Typical	4	67%
	Perceived Limitations	Typical	3	83%
Outcome expectancies				
	Negative outcome expectancies	Typical	4	67%
	Hope for future relationships	General	6	100%

### 4.1. Attitudes about intimacy

This domain represents a synthesis of information from the initial prompt asking about important relationships and why they were important and other information that developed naturally throughout the interviews. Attitudes about intimacy varied across participants. Categories that emerged were core values related to intimacy, barriers to intimacy, and non-romantic intimacy.

#### 4.1.1. Attitudes about intimacy: core values (6, general)

Every participant endorsed what this research has labeled as core values of intimacy and gave insight into what was most important for intimate relationship development. The most endorsed core-value was *Shared Experiences* (4,typical). Attitudes ranged from the joys of dating and sharing experiences, “Just being able to connect with someone… even if it was just for an evening.” to an emphasis on the shared experiences of long-term relationships.

Participants endorsed what was coded as the sub-category of *Shared Values* (4,typical). These values often related to their partners sharing their values about intimacy. One participant emphasized openness to disability as a “…lens to see the world through. How you define beauty, how is beauty important, and do you have people that see past the surface?” Other notable shared values included honesty, trust, openness to intimacy, and communication. Two participants talked at great length about their openness to intimacy. Both commented how their openness has contributed to their successes in intimate relationship development. One said her successes were due to her “…openness and willingness to do intimate things,” while the other said, “…having that level of confidence in my sexuality and my sexual preferences, also would be a success.”

Attraction (2,variant) as a sub-category highlighted both the need for emotional and physical attraction in intimate relationships. One person denounced physical attraction and went so far as to call it “shallow,” while the other person emphasized that relationships that were purely emotional were lost because, “You kind of have to be attracted to someone in other ways that are not in the emotional.”

#### 4.1.2. Attitudes about intimacy: non-romantic intimacy (4, typical)

While the project’s initial intent was to develop a better understanding of romantic intimacy, the definition of intimacy was left broad to capture the women’s experiences better. Four women endorsed the sub-categories, *Family and Friend Intimacy* (4,typical) and *Conflicting Roles of Intimacy* (4,typical).

Four women recognized friendship and familial intimacy as having a significant impact on their attitudes, both romantic and non-romantic. Those relationships provided, “grounded, consistent, and respectful mutual relationship[s].” The women focused on the comfort and the value of deep emotional connections with friends or family. “It’s really important that you can share your heart…I think that’s when you have your most intimate, wonderful relationships.”

Three women focused on *Conflicting Roles of Intimacy* (3,typical). This category focused on the forced intimacy of caregivers and how that role can conflict with romantic intimacy. One participant shared how her, and likely other’s attitudes about intimacy, were shaped by having a caregiver, and the other two recounted difficulty for partners switching roles between caretaker intimacy and romantic intimacy

*It bothers me when my partner of 20 years has got to help me to the bathroom. I don’t always want her to have to be in that position. That’s not sexy. It’s super intimate, but there is nothing sexy about going to the can with me*.

### 4.2. Barriers to intimate relationship development

All of the participants identified significant barriers to intimacy; specifically, the barriers mentioned were condensed down to 3 distinct sub-categories, *stigmatizing effects of disability* (6,general)*, physical barriers to intimate relationship development* (4,typical)*, and perceived barriers* (3).

#### 4.2.1. Societal barriers to intimacy (6, general)

All of the women surveyed people mentioned the stigmatizing effects of disability as a societal barrier to intimacy.

*The idea of having a physical disability creates celibacy or creates a lack of aptitude when it comes to sexual activities or a lack of enjoyment when it comes to sexual activities. I don’t think any of those are true*.

Some respondents recounted how the perceptions of others shape their dating lives. One respondent believes that people often do not consider her as a romantic partner because of her disability. “I think they ‘friend zone’ me relatively quickly because a lot of people that I date…have limited experience with the disability community.” Another respondent reported feeling an overall pressure and vulnerability related to her disability and how the able-bodied world interacts with women with disabilities.*”* I feel vulnerable to the judgment of other people, to microaggressions, and to being framed according to the language and the stereotypes.” Several others referred to people not viewing them as dateable or intimate beings. One even recounted being viewed as a means of satisfying curiosity about how people with disabilities have sex. Another participant described how her perceived fragility is a limiting factor in her development of romantic and non-romantic intimate relationships. “Touching, because of my wheelchair, I feel that most everybody, even my family members, are sometimes very uncomfortable hugging, touching; they’re afraid it might hurt me.”

#### 4.2.2. Physical barriers to intimacy (4, typical)

Four participants shared struggles related to physical barriers to intimacy, capturing the various biological and functional limitations disability poses on intimate relationship development. For instance, disability causes not only physical distance, “I’ve always wanted to hold someone’s hand and walk, but because I have a walker,” but also lack of spontaneity in relationships, “We can’t necessarily make out in a car because the fact of my disability makes it awkward and difficult to do that.”

#### 4.2.3. Perceived limitations (3, typical)

There was some overlap with the sub-category *Stigma* and sub-category *Perceived Limitations* (3,typical), explicitly discussing how disability affects perceptions of others who are not disabled. One participant acknowledged her inability to communicate her needs effectively and attributes that to assumptions about her partner’s perception of her disability. *“*I’ll just assume that the other person won’t get it, and I won’t talk about it, and I’ll just shut down.” Similarly, the same participant focused on overcoming these *Perceived Limitations* as a process “…That has been more of a barrier to me than the people themselves, or the way that they treat me.”

### 4.3. Outcome expectancy—hope for the future

The two categories that emerged from this domain are *Hope for Future Relationships* (6,general) and *Negative Outcome Expectancy* (4,typical). The two categories are discussed below in reverse order as the survey’s final question was about hope for the future.

#### 4.3.1. Outcome expectancy: negative outcome expectancy (4, typical)

This category mostly centered around past formative experiences that affected each person’s outcome expectancy. One participant had a negative outcome expectancy for dating other people with disabilities because of her previous experience. Another reflected that she had her heart broken too many times to have hope for an intimate relationship “it is no longer something I have been able to do is be intimate because I’ve gotten my heart broken way too many times.” Another focused on messages that she had gotten from her family early in life that she is still combatting, “Those kinds of rejections were again validating maybe mom was right.”

#### 4.3.2. Outcome expectancy: hope for the future (6, general)

The interview concluded with a question about hope for the future. Each of the women articulated either what they wanted from themselves, what they wanted from their partners, or what they wanted in intimate friendships. One participant hoped for the relationship she had now but in the future. At the same time, others talked about matching partner values and relationships. One person wanted a relationship where she “*can feel comfortable being vulnerable and sharing the parts of myself that are most complex, and a lot of times that means my disability and the way that it affects my identity.”* Still, another participant hoped for a healthy romantic relationship, both emotionally and sexually, despite the work that comes with longer-term relationships.

Concerning *Non-Romantic Relationships*, one participant specifically mentioned intimate friendships. She is confident that she will develop more intimate friendships, and while she had mentioned that she felt too old for romantic relationships earlier in the interview, she said, “I’ve *never been willing to turn off the idea that I will find someone that might find me sexually attractive or intimately attractive.”*

## 5. Discussion and implications

The research may have utility for both practitioners as well as people with disabilities themselves. Though broad in focus and conducted with a small group, this study yielded interesting results relative to the development of intimate relationships, including information on delayed intimate relationships, sexual self-concept, and availability of relationships.

### 5.1. Delayed intimate relationships

Several common societal beliefs prevent people with disabilities from exploring and learning about their sexuality leading to lesser exposure to the same psychosexual developmental opportunities as people without disabilities (39). Several clients stated that they didn’t have opportunities to date or develop romantically intimate relationships before leaving high school, which is supported by the literature (40). Attitudes about sexuality and disability can lead to the internalization of negative attitudes and beliefs (13). As shown through these interviews, these attitudes derive from a multitude of places, such as formative family experiences and the media. It can be highly detrimental to the self-esteem of people with disabilities to be considered unattractive and have essentially a nonsexual status in society (6). Isolation from peers and not being perceived as suitable for dating (41) seemed to have attributed to a vague sense of sexual identity (42) for several of the women surveyed.

#### 5.1.1. Sexual self-concept

People with disabilities are not readily included in societal concepts of sexuality, leading to a devaluation of sexual identity. Several participants identified family values as playing a role in how they conceptualize themselves. One participant focused on her intersecting identities as a person with a disability and her cultural identity as of Caribbean descent. Disability was seen as a weakness in her culture and a negative reflection on her family. Another participant discussed how her other identities (e.g., student, worker, friend) took priority over relationship development. This holds with Bishop’s Disability Centrality model that discusses quality of life as moderated by domain importance (43). For that particular participant, intimate relationships shifted to lower importance when compared to her other identities and disability management.

#### 5.1.2. Sexual self-efficacy

Women who reported positive self-efficacy seemingly engaged more in intimate relationship development tasks and seemed better able to cope with rejection. Conversely, individuals who appeared to have low self-efficacy for intimate relationship development had fewer opportunities and seemed to give up more quickly, which served to lower self-efficacy. Thus, participants’ self-efficacy, whether positive and negative, seemed to dictate their sense of identity and behaviors towards intimate relationships (42).

### 5.2. Salient aspects of intimacy and sexuality

Some of the most interesting results related to participants’ perceptions of the most critical aspects of intimacy and sexuality. Of particular interest for this and possibly future research were the areas of core values and outcome expectancy. Each participant shared their insight as to what was essential for them with regards to intimate relationship development. The resulting themes were centered around openness to experience, shared values, and validating relationships.

#### 5.2.1. Openness to experience

A broader conceptualization of intimacy to include romantic and non-romantic intimacy allows for a view of intimacy as a normative part of overall well-being for people with disabilities. Encouraging positivity regarding intimacy has been shown to have important implications for the overall health and quality of life (44). Participants were careful to distinguish intimacy as connectedness and broadly relational and not as just sexually related.

#### 5.2.2. Shared values

Of the many values of importance to individuals with disabilities listed, the most ubiquitous in this study was communication. Communication was detailed as a strength, a limitation, and an essential quality in a future intimate relationship. Communication with respect to intimate relationships may be directly linked to relationship self-efficacy. Other aspects of core values that were identified were honesty, trust, comfort, and attraction.

#### 5.2.3. Validating relationships

Another central theme that was present was validating relationships and shared experiences. Several participants shared experiences of validating or invalidating relationships with peers, family, and friends. Those validating and invalidating relationships influenced many aspects of participants’ lives, including their disability identity, self-efficacy, self-concept concerning relationships, and their outcome expectations.

### 5.3. Challenges and facilitators to intimate relationship development

This project’s larger scope was better to understand barriers and facilitators to intimate relationship development. Psychosocial research often focuses primarily on barriers. The intent of this research was to focus more on the facilitators to intimate relationship development.

#### 5.3.1. Barriers

Four out of six participants said that disability, itself, did not present any barriers or insurmountable barriers to developing relationships. This research supported past investigations that enumerated the multiple barriers people with disabilities face within any psychosocial interactions. There were a few barriers that bear mentioning in that they help form a richer picture of intimate relationship development for women with physical disabilities and the hardships they endure.

Women in the study talked about specific barriers to intimacy that shaped how they perceive and navigate intimate relationships. Barriers mentioned were physical limitations of disability that included age and functionality, and the physical logistics of spontaneity. Some women reported that society perceived them as frail and lacking desire based on their disability. These perceptions are often perpetuated by myths or stigma about disability (4). Of course, stigma plays a significant role in shaping how these women navigate intimate relationship development. Several of the women focused on negative societal barriers and the stigmatizing effects of disability. These findings are supported by the literature, which suggests that these women are perceived as less worthy, less valuable, and less desirable as intimate partners (14, 15). They reported avoidance, rejection, and patronization. Several mentioned specific formative negative experiences as a result of stigma that influenced their identity, their self-concept, their self-efficacy, and their outcome expectations.

#### 5.3.2. Facilitators

There were three standout themes about facilitators of intimate relationships: openness, communication capacity, and self-concept. The women were asked about some of their personal strengths and what sustained their relationships. One of the most salient themes was openness. The women that were more open to new experiences and risk-taking had well-developed interpersonal skills and communication skills.

In the same vein, communication skills were a repeated theme throughout the research process. For instance, one participant noted a communication skill deficit hindered her relationships, while another participant, who was reportedly more successful with relationships, noted it as one of her strengths. The reportedly more successful women were confident enough to initiate relationships and confident in expressing and advocating for their wants and needs.

The third salient theme that was a facilitator of intimate relationships was self-concept and self-efficacy. Women who were more confident in their identities as people with disabilities, as measured by willingness to disclose disability, were more apt to have higher intimate relationship self-efficacy. Self-concept and disability identity seem to be related to specific formative experiences. Specific formative experiences such as the imposition of family values related to disability or specific experiences related to disability may alter individuals’ attachment patterns.

The results provide some insight into the question, “What makes some people more successful than others at developing intimate relationships?” In any community, there are people with uncommon but successful strategies that help them find better solutions to problems than their peers. This research has sought to label facilitators of intimate relationships for people with physical disabilities. It is the hope that future research will focus more specifically on this severely neglected area that is of the utmost importance to people with disabilities**.**

### 5.4. Limitations

The implications of this study must be considered within the context of its limitations. First, the number of participants was roughly half of the recommended participants by Hill and colleagues (2005) (32) due to financial restraints, limiting our ability to achieve saturation on our qualitative study. Second, it is unlikely that this group’s views and behaviors include the wide range of viewpoints of all women or all people with visible physical disabilities. This study’s findings are representative of women with physical disabilities and may not extend to cover all experiences of women with other types of disabilities. This was done intentionally to control for potential stigma visible disabilities may cause as well as other disability experiences. Additionally, we acknowledge the potential for different categorizations of qualitative data that could yield different insights and have chosen our current format using CQR with 3 researchers and an auditor for a nuanced understanding of factors impacting intimate relationships for these women. Finally, the subject material itself is a limitation. While it was consented to, the subject material may have yielded responses that may have been subject to bias from the discomfort of participants in discussing other intimacy issues. Despite these limitations, the present study provides a rich description of these women’s experiences about intimate relationship development.

### 5.5. Suggestions for future research and practice

The present study’s limitations are best addressed by future research. Future research is planned to explore more specific aspects of this topic, such as the influential factors of intimacy. Given the absence of current research focused on intimate relationship development, the literature would benefit from replicating the current study. Such research would help to solidify or disconfirm the current findings. Ideally, replication of the study would include more women so a more accurate picture can be gained of the effects of age, specific disability, and other interpersonal characteristics. The inclusion of more people, and eventually men, would highlight the inter and intrapersonal factors that facilitate intimate relationship development.

Expanding research in this area may serve as an initial point for new interventions that seek to increase relational capacities and sexual well-being, reduce sexual risk-taking, and enhance the intimate relationships of people with disabilities. It may seem obvious that people with disabilities may show deficits in areas of sexual well-being. It is the responsibility of rehabilitation professionals, educators, and parents to assist with the reduction of sexual risk-taking and enhance the intimate relationships of people with disabilities. A research study by Kazukauskas and Lam (21) indicated that roughly 40% of rehabilitation counselors did not receive any sexuality-related training at all in graduate school. Integration of more intimacy, sexuality, and relationship training within the rehabilitation curriculum will be a boon to counselors as well as clients and fill a gap in training that has long been an issue. Intimacy-related goal planning, as part of integrated care plans, may help all people struggling with disability-related intimacy issues but may be especially helpful to people who acquire disabilities and older individuals within the community who have experienced more significant stigma related to disability and intimacy.

Several participants endorsed the importance of mentoring and the development of interpersonal skills through either mentorship or specific classes. These programs, especially if initiated early in school or adjunct to typical schooling, may facilitate intimate relationship empowerment. Empowerment has been argued as the opposite of self-stigma and is defined as having control over one’s treatment and life (45). A strong sense of personal empowerment is highly correlated with high self-efficacy and self-esteem. Self-esteem has been identified as a moderator for psychological responses to interpersonal rejection (46). Communities may foster personal empowerment and pride through programming that provides access to education, normalization of experiences, and community mentors.

It would stand to reason that exposure to other adults with disabilities early on would reduce self-stigma, as well as imposed familial stigma (47). Hope is strongly correlated with high optimism, self-esteem, perceived control, and problem-solving abilities. Literature indicates that hope levels are responsive to targeted interventions that provide goal-directed thinking, adaptive coping, and focus on attachment-related outcomes (48). Rehabilitation and community settings may provide settings to implement hope theory-based interventions (49) and apply them directly to intimacy-related constructs such as sexual communication self-efficacy, coping with rejection, and intimacy-related goal planning.

## 6. Conclusion

This Consensual Qualitative Research project aimed to gain a better understanding of barriers and facilitators to intimate relationship development. Results indicated that women with physical disabilities attitudes about intimacy include barriers to intimacy and core values, that limitations of disability and disability identity are impacted in varying degrees by partners, other relationships, and barriers. In terms of outcomes expectancy, our participants had overall hope for future relationships and strong self-efficacy regarding their own personal strengths. Finally self-concept was impacted by the effects of the physical disability the women had, including disability stigma.

This researcher was asked by participants throughout the process, “What can be done?” and the answer is a lot. Rehabilitation professionals are responsible for normalizing intimacy. That can be accomplished simply by asking people about it. Rehabilitation professionals can be strong advocates for ways that people with disabilities can gain the social skills and self-confidence necessary to combat the stigma present within our society.

## Data Availability

The datasets presented in this article are not readily available. Requests to access the datasets should be directed to derek ruiz, derek.ruiz@sus.edu.
