# Supplementary material for: The pursuit of intimacy: intimate relationship development for women with physical disabilities
Source: Front Rehabil Sci. 2023 Aug 4;4:1070501. doi: 10.3389/fresc.2023.1070501 (PMC10441775; doi:10.3389/fresc.2023.1070501)
Supplement: Supplementary file 1 [file Table1.docx]

Supplementary Material

The Pursuit of Intimacy: Intimate Relationship Development for Women with Physical Disabilities

Derek Ruiz, Ph.D., CRC, LPC1*, Alexandra Kriofske Mainella Ph.D., CRC*, David Rosenthal Ph.D., CRC3

*** Correspondence:**Derek Ruiz
derek.ruiz@sus.edu

# Supplementary Data

**Interview Protocol:**

This study is about people with disabilities establishing meaningful and intimate relationships and some of the facilitators, barriers, and significant challenges in establishing, maintaining and developing those relationships. I will be asking you questions about intimacy related to dating and love. Are you comfortable if I ask you these questions?

1. Will you begin by telling me a little bit about yourself and about your disability if you are comfortable.
2. Could you describe what you would consider some of the important intimate relationships you’ve had in your life?
   1. Can you tell me what aspects of intimate relationships are most important to you and why they are important?
   2. *[If previous relationship]* Can you tell me a little bit about what sustained that/those relationships? Can you tell me about what limited the relationship?
   3. *[If current relationships]* What are your hopes for this intimate relationship in the future?
3. How would you describe your confidence in developing intimate relationships? Why?
   1. Please describe your ability to develop meaningful intimate relationships.
4. Can you tell me a little bit about the experiences you have you had with dating, both positive and negative?
   1. How have your experiences affected your intimate relationships?
   2. How have your experiences views about yourself affected your intimate relationships?
   3. What barriers, if any, has your disability caused in dating and developing intimate relationships?
5. Could you describe what has been most successful for you with regards to forming intimate relationships?
   1. Are there personal strengths that have contributed to your success in developing intimate relationships?
   2. Are there any personal challenges that have gotten in the way of your success with intimate relationships?
6. What are your hopes for intimate relationships in the future?
   1. Describe what an ideal intimate relationship would look like for you?
7. Can you tell me a little bit about how you use social media and social networking?
   1. How do social media and social networking play into development of intimate relationships?
